# Anionic Polymerization of *β*-Butyrolactone Initiated with Sodium Phenoxides. The Effect of the Initiator Basicity/Nucleophilicity on the ROP Mechanism

**DOI:** 10.3390/polym11071221

**Published:** 2019-07-22

**Authors:** Adrian Domiński, Tomasz Konieczny, Magdalena Zięba, Magdalena Klim, Piotr Kurcok

**Affiliations:** 1 Centre of Polymer and Carbon Materials, Polish Academy of Sciences, 34, M. Curie-Sklodowskiej St., 41-819 Zabrze, Poland; 2 Faculty of Mathematics and Natural Science, Jan Dlugosz University in Czestochowa, 13/15, Armii Krajowej Ave., 42-200 Czestochowa, Poland

**Keywords:** *β*-butyrolactone, anionic ring-opening polymerization, phenoxides, polymerization mechanism, *β*-lactones, polyhydroxybutyrate

## Abstract

It was shown that selected sodium phenoxide derivatives with different basicity and nucleophilicity, such as sodium *p*-nitrophenoxide, *p*-chlorophenoxide, 1-napthoxide, phenoxide and *p*-methoxyphenoxide, are effective initiators in anionic ring-opening polymerization (AROP) of *β*-butyrolactone in mild conditions. It was found that phenoxides as initiators in anionic ring-opening polymerization of *β*-butyrolactone behave as strong nucleophiles, or weak nucleophiles, as well as Brønsted bases. The resulting polyesters possessing hydroxy, phenoxy and crotonate initial groups are formed respectively by the attack of phenoxide anion at (i) C2 followed by an elimination reaction with hydroxide formation, (ii) C4 and (iii) abstraction of acidic proton at C3. The obtained poly(3-hydroxybutyrate) possesses carboxylate growing species. The ratio of the observed initial groups strongly depends on the basicity and nucleophilicity of the sodium phenoxide derivative used as initiator. The proposed mechanism of this polymerization describes the reactions leading to formation of observed end groups. Moreover, the possibility of formation of a crotonate group during the propagation step of this polymerization is also discussed.

## 1. Introduction

Poly(β-hydroxyalkanoate)s (PHAs) are biodegradable and biocompatible aliphatic polyesters [1], considered to be future “green” materials for biomedical, food package or drug delivery applications [2,3,4]. Several PHAs are produced by many different microorganisms [5], but the most well-known is poly([R]-3-hydroxybutyrate) (PHB). Isotactic PHB is a biopolymer produced by microorganisms and used as an energy and carbon source [6]. Synthetic and natural poly(3-hydroxybutyrate)s are very popular for biomedical applications due to their biodegradation and biocompatibility [2,3,4,7]. Poly([R]-3-hydroxybutyrate) is synthesized by biotechnological processes, applying bacterial fermentation of substrates such as sucrose, corn, cane sugar, etc. [8], or as a result of a chemical reaction, i.e., polymerization of *β*-butyrolactone (4-methyl-2-oxetanone, BL), using coordination, cationic or anionic ring-opening polymerization [9]. 

Coordination polymerization allows for obtaining atactic, syndiotactic and perfectly isotactic PHB depending on the type of coordination catalyst used as well as the way of conducting the reaction. The most common coordination catalysts are based on metals such as aluminum, tin, yttrium, zinc and lanthane [10,11,12,13]. The polymerization of *β*-butyrolactone can also be carried out using cationic initiators such as trifluoromethanesulfonic acid or hexafluorophosphate. However, the cationic polymerization is characterized by a low molar mass of the resulting polymer, which seems to be unattractive [14,15]. 

Anionic ring-opening polymerization (AROP) of BL is considered one of the most interesting methods, due to the simplicity of the reaction, lack of toxicity of the resulting polymer, inexpensive catalysts and the possibility of preparation of tailor-made materials. Additionally, AROP allows for obtaining poly(3-hydroxybutyrate) with the desired microstructure depending on the absolute configuration of the monomer used (R, S or a R,S mixture) and the method of carrying out the reaction [16,17,18]. Since the 1990s, many scientists have described the mechanism of anionic ring-opening polymerization of *β*-butyrolactone with various initiators, i.e., alkali metal alkoxides, carboxylates, naphthalenides, hydrides or basic organocatalysts. These studies have shown that the initiation step of *β*-butyrolactone polymerization strongly depends on the type of anionic initiator used [16,18,19,20].

**Figure 1:**
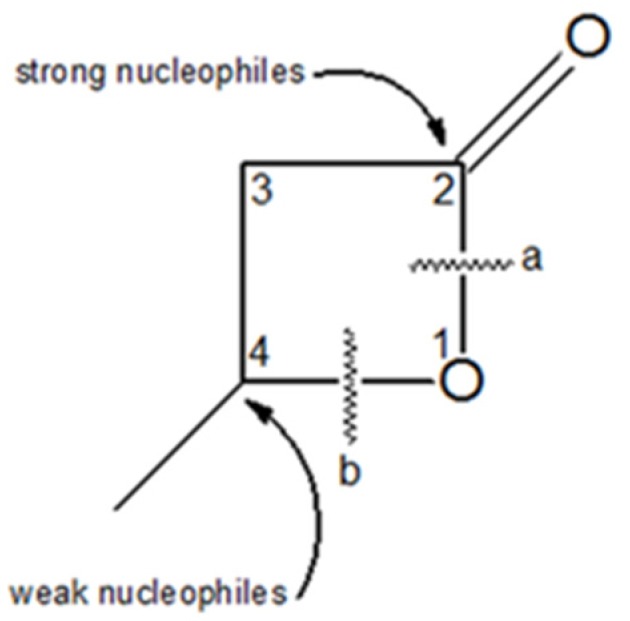
*β*-butyrolactone molecule with carbon description and possible nucleophile attack positions in anionic BL polymerization.

When alkali metal naphthalenide is used in BL anionic polymerization, the initiation of *β*-lactone ring-opening polymerization occurs as a result of exclusive deprotonation at the C3 carbon of the BL ring by the naphthalenide. The unstable enolate formed in this reaction is rearranged to the crotonic acid salt. The unsaturated acid salt formed is a real initiator of the polymerization, where centers of propagation are carboxylate ions [19,21]. The same situation occurs in the case of applying an alkali metal hydride as an initiator in the BL polymerization process [22,23]. Recently, basic organocatalysts like guanidine (TBD), amidine (DBU) and phosphazene (BEMP) were investigated as *β*-lactone initiators due to efficient polymerization of BL in mild conditions [24,25]. In addition, Coulembier et al. [26] proved that polymerization of BL initiated with TBD mainly proceeds with initial deprotonation of *β*-lactone and crotonate with a protonated base counterion (e.g., TBDH^+^) formed as a real initiator of this polymerization [20,26]. In the case of the *β*-lactone ring opening with potential covalent linkage initiator-3-hydroxyalkanoate unit formation, there are two main classes of initiators described: weak nucleophiles such as carboxylic acid salts and strong nucleophiles, i.e., alkali metal alkoxides (see Figure 1).

In *β*-lactones AROP, the initiation reaction by a weak nucleophile, e.g., a salt of carboxylic acids, proceeds according to the S_N_2 mechanism. The initiator attacks the C4 carbon of the *β*-lactone ring with alkyl-oxygen bond cleavage (**b** in Figure 1). The carboxylate ion formed in this reaction is a center of the chain growth. The activity of the initiator and the chain propagation center strongly depend on the cation–anion interactions, the size of the counterion and the solvent polarity. The activity of the initiating system can be easily manipulated [27,28,29,30,31]. When the polymerization initiator is an alkali metal alkoxide, which is a strong base and a strong nucleophile, the reaction proceeds according to the addition–elimination mechanism. The initiator attack occurs at carbonyl carbon C2 and the ring opens in the acyl-oxygen position (**a** in Figure 1). However, the polymer chain growing center type (alcoholate or carboxylate) strongly depends on the *β*-lactone structure, i.e., the presence of substituents at the C3 carbon of the lactone ring [32,33]. In the case of *β*-butyrolactone, the crotonate and 3-hydroxybutyrate salts generated in the preinitiation step are the real initiators in this anionic ROP. The chain growth takes place entirely via carboxylate active centers, which leads to the formation of poly([R,S]-3-hydroxybutyrate) containing hydroxy and crotonate initial groups and carboxylic end groups [34,35,36,37].

The aim of the present work was to investigate the mechanism of anionic ring-opening polymerization of BL initiated with selected sodium phenoxides, i.e., weak nucleophiles/bases with the alkoxide structure of active species. Particular attention was paid to the initiation of polymerization, i.e., the reaction of *β*-lactone molecule with selected phenoxides possessing different basicity/nucleophilicity.

## 2. Materials and Methods

### 2.1. Materials

β-Butyrolactone (+98% Aldrich, Steinheim, Germany) was purified as described in [37]. Phenol and 1-naphthol (both +98% POCH, Gliwice, Poland) were purified by sublimation before use. *p*-Nitrophenol, *p*-chlorophenol and *p*-methoxyphenol (all +99%, Aldrich, Steinheim, Germany) were used as received. All sodium phenoxides were prepared by potentiometric titration of the respective phenol water solution with 1M NaOH. Next the water was stripped-off from the reaction mixture and all obtained salts were dried under vacuum at 60 °C for 48 h. 18-Crown-6 ether (+99% Aldrich, Steinheim, Germany) was dried under vacuum at 55 °C overnight before use. Dowex 50WX8 (Aldrich, Steinheim, Germany) was washed with dry THF before use. DMSO (POCH, Gliwice, Poland) was distilled under reduced pressure over CaH_2_ followed by two repetitions of reduced pressure distillation over BaO. THF (POCH, Gliwice, Poland) was distilled over a potassium-sodium alloy. CHCl_3_, hexane, KMnO_4_, NaOH (all POCH, Gliwice, Poland) were used as received.

### 2.2. Methods

^1^H NMR (600 MHz) spectra were recorded using a Bruker-Avance II 600 MHz (Fremont, CA, USA) with Ultrashield Plus Magnets at r.t. in CDCl_3_ or DMSO-*d*_6_ with tetramethylsilane (TMS) as an internal standard. 

Size exclusion chromatography (SEC) was conducted in CHCl_3_ at 35 °C with a flow rate of 1 mL/min, using a Spectra-Physic 8800 gel-permeation chromatograph (Santa Clara, CA, USA), equipped with a PL-gel 5 mm MIXED-E ultra-high efficiency column and Shodex SE 61 differential refractive index detector. Polystyrene standards with a low dispersity mass distribution (*M*_p_ ranged from 162 to 19,760 g·mol^−1^) were used to generate a calibration curve.

Electrospray mass spectrometry (ESI-MS^n^) was performed using a Finnigan LCQ ion trap mass spectrometer (Finnigan, San Jose, CA, USA). The poly(3-hydroxybutyrate) samples were dissolved in methanol/chloroform mixture (1:1, *v*/*v*). The solutions were introduced into the ESI source by continuous infusion at 7 μL·min^−1^ using the instrument’s syringe pump. The LCQ ESI source was set to an operating voltage of 4.5 kV and the capillary heater was set to 200 °C. Nitrogen was used as the nebulizing gas. Helium was used as the collision and damping gas in the ion trap. 

GC-MS was performed using an Agilent gas chromatograph 7890C (HP-5 MS capillary column, 30 m × 0.25 mm × 0.25 μm), coupled with an Agilent mass spectrometer with EI ionization (70 eV). Products were identified using the NIST-EPA-NIH Mass Spectral Database.

Fourier transform infrared (FTIR) spectra were recorded on a Jasco FT-IR 6700 spectrometer (Tokyo, Japan) equipped with a MultiLoop-MIR Silver Halide Probe Set in the region between 2000 and 800 cm^−1^ with 128 scans.

### 2.3. General Polymerization Procedure

The polymerizations were performed in an anhydrous atmosphere (glove-box, H_2_O < 1 ppm). Ring-opening polymerization of *β*-butyrolactone initiated with selected phenoxide was carried out in dry DMSO (or in THF with the addition of 18-C-6 crown ether in molar ratio 1:1 as a cation-complexing agent) at the monomer concentration equal to 1 mol/L or 8 mol/L at room temperature. Conversion of the monomer was monitored by using FTIR spectroscopy (disappearance of the band derived from the carbonyl group *β*-butyrolactone at 1820 cm^−1^). The polymerization reaction was quenched by acidification with Dowex 50WX8. The obtained polymer was precipitated in cold hexane, dried under vacuum to constant weight and characterized by ^1^H NMR, ESI-MS^n^ and SEC techniques. The filtrate obtained after polymer precipitation was analyzed by using the GC-MS technique. 

## 3. Results and Discussion

In the present work, it is shown that various sodium phenoxides obtained by the use of phenols with different pKa values, such as: *p*-nitrophenol, *p*-chlorophenol, 1-napthol, phenol, and *p*-methoxyphenol, are effective initiators of *β*-butyrolactone AROP in mild conditions. As mentioned in the Introduction, alkoxides attack at the C2 carbon, while the carboxylic acid salts at the C4 one in the BL monomer. In both cases, there are significant differences in the basicity and nucleophilicity of both types of initiators and different starting groups were observed in the polymer chain as a result. It prompted us to investigate the polymerization of BL initiated with selected sodium phenoxides, i.e., a group of compounds that are weak nucleophiles, but with an alkoxide structure. The basicity/nucleophilicity of these initiators can be easy adjusted by application of an appropriate substituent in the phenoxide ring. In the anionic polymerization of BL initiated with alkali metal alkoxides and carboxylates, usually activation of an initiator is required. In this study, applied sodium phenoxides were activated by sodium counterion complexation with crown ether when the polymerization was carried out in THF or by using the solvent strongly activating anionic initiator i.e., DMSO [27]. Most of AROP experiments were performed in DMSO. Therefore, the basicity of the initiators used, based on the pKa of selected phenols (in DMSO) [38], was as follows: *p*-NO_2_PhONa < *p*-ClPhONa < NapthONa < PhONa < *p*-CH_3_OPhONa).

The results presented in Table 1 show that all five selected phenoxides initiated polymerization efficiently, and PHB with a controlled number average molar mass up to 10,000 g/mol with an acceptable level of dispersity (Đ c.a. 1.2) was synthesized. However, it is very important in such polymerization to select the proper conditions of the process. In entries 6–10 (Table 1), the measured M_nSEC_ indicates the loss of PHB molar mass control. This can be explained by the competition of propagation and side reactions, e.g., chain transfer to the polymer proceeding via the E1cB (elimination unimolecular conjugate base) mechanism [39,40], resulting in polymer degradation. Due to low initial monomer concentration (1mol/L), the reaction rate is slow and transfer to the polymer can be competed with propagation. However, when the monomer concentration was increased up to 8 mol/L, it was possible to obtain the polymer with the expected molar mass (see entries 11 and 12). Moreover, chain transfer to the monomer was also possible in this polymerization, although the obtained results seem to indicate that in fact transfer to the polymer is responsible for the significant reduction in molar mass observed in this polymerization (see entries 6–10).

**Figure 2:**
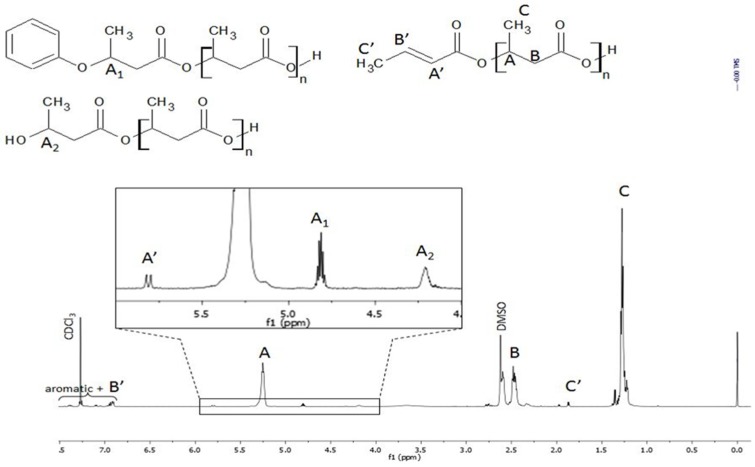
^1^H NMR spectrum (CDCl_3_, 600MHz) of poly(3-hydroxybutyrate) obtained via AROP of BL initiated with sodium phenoxide.

Results of ^1^H NMR and ESI-MS^n^ analyses revealed that the poly(3-hydroxybutyrate)s obtained in AROP initiated with the selected phenoxides possess carboxyl end groups and three different types of initial groups: crotonate, phenoxy and hydroxy, occurring in different ratios, depending on the phenoxide used. A typical ^1^H NMR spectrum of low molar mass PHB obtained in the polymerization initiated with sodium phenoxide shows the polymer chain characteristic signals: (CH_3_ at δ = 1.28 ppm, CH_2_ = 2.5 ppm, CH = 5.25 ppm) and signals corresponding to initial groups: (HO–C**H** at δ = 4.2 pm, PhO–C**H** at 4.8 ppm and CH_3_CH=C**H** at 5.8 ppm) (Figure 2).

**Figure 3:**
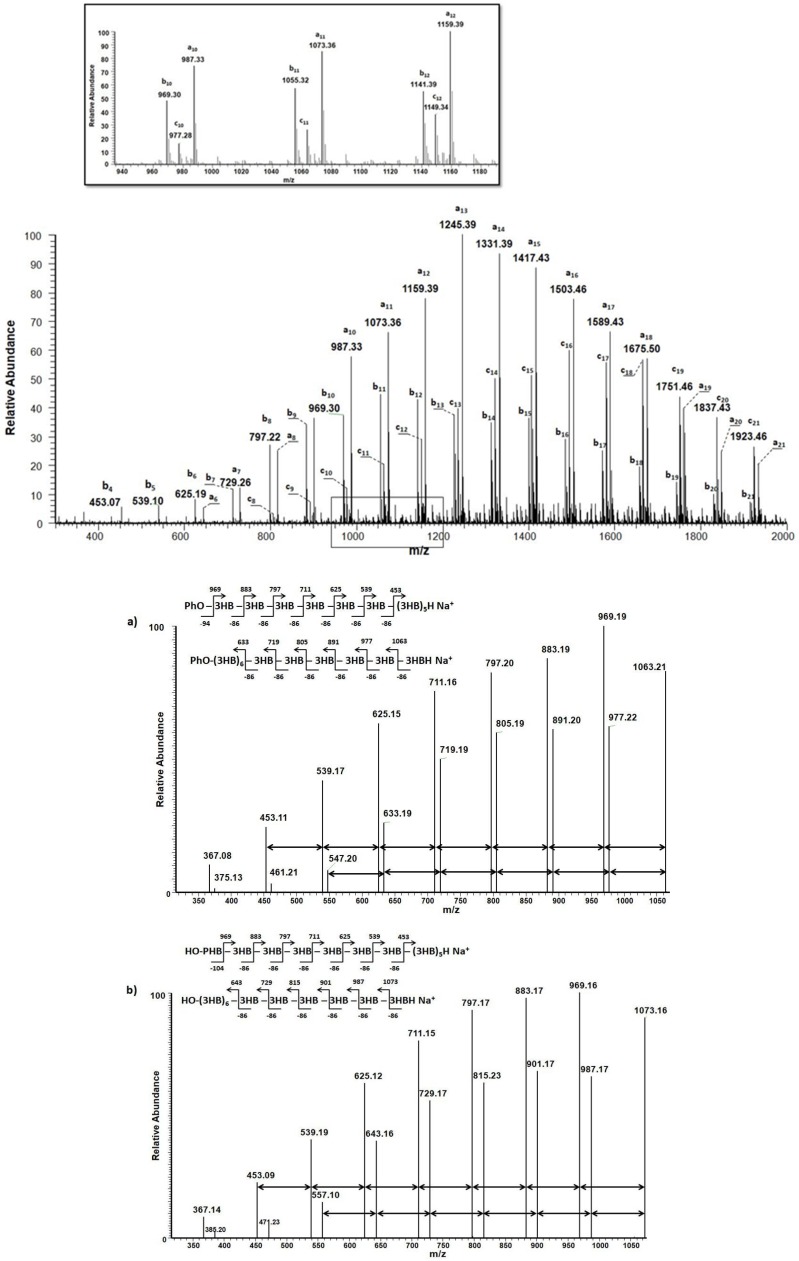

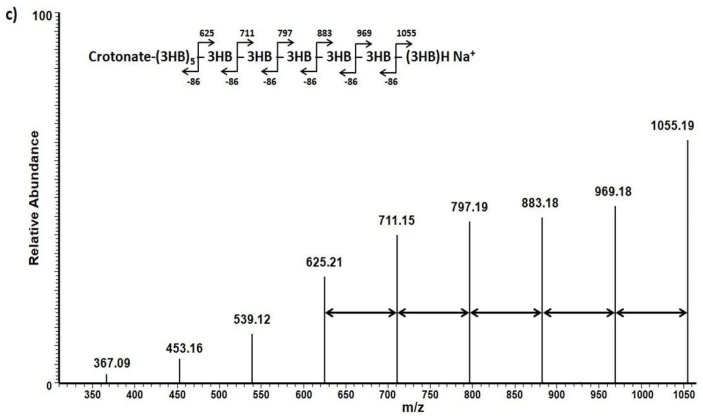
ESI-MS (positive-ion mode) of poly(3-hydroxybutyrate) obtained via AROP of BL initiated with sodium phenoxide; series a_n_—sodium adducts of *α*-hydroxy-*ω*-carboxylate-PHB chains (ESI-MS^2^ fragmentation spectrum (positive-ion mode) of corresponding parent ion *m/z* 1073 is shown in Figure 3b); series b_n_—sodium adducts of *α*-crotonate-*ω*-carboxylate-PHB chains (ESI-MS^2^ fragmentation spectrum (positive-ion mode) of corresponding parent ion *m/z* 1055 is shown in Figure 3c), series c_n_—*α*-phenoxy-*ω*-carboxylate-PHB chains (ESI-MS^2^ fragmentation spectrum (positive-ion mode) of corresponding parent ion *m/z* 1063 is shown in Figure 3a). Index n corresponds to the number of PHB repeating units.

Analysis of an ESI-MS^n^ spectra (Figure 3) of PHB obtained in the reaction initiated with sodium phenoxide shows three different signals populations in the investigated polymer. The most intensive population (c_n_) corresponds to adducts of α-hydroxy-ω-carboxylate-PHB chains with Na^+^, e.g., *m/z* = 1073.2 g/mol for n = 11. The second signal population (a_n_) assigned to α-crotonate-ω-carboxylate-PHB chain adducts with Na^+^ (e.g., the peak with *m/z* = 1141.2 for n = 13) and finally, the population (b_n_) corresponds to α-phenoxy-ω-carboxylate-PHB chain adducts with Na^+^ (*m/z* = 1063.2 for n = 11). These results are clear evidence that the polyester obtained contains molecules with crotonate, phenoxy and hydroxy initial groups and carboxyl end groups.

**Scheme 1:**
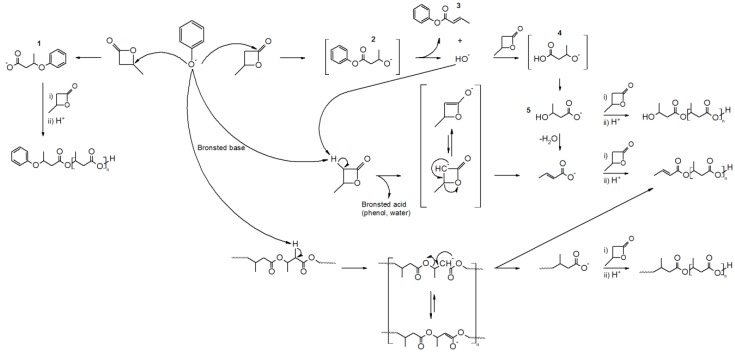
AROP of *β*-butyrolactone initiated with sodium phenoxide (for simplicity, the Na^+^ counterion is omitted).

The nature of PHB chain initial groups confirmed by ^1^H NMR and ESI-MS indicates that, at the initiation step of AROP, ring-opening of the BL monomer occurs at the acyl-oxygen and alkyl-oxygen positions, i.e., similar to BL polymerization initiated with weak nucleophiles (S_N_2 at carbon C4) as well as strong nucleophiles (addition–elimination mechanism) (Scheme 1).

The phenoxide anion reacts as a nucleophile in a substitution reaction (i) or as base in an elimination one (ii). The substitution reaction occurs at the C4 and C2 carbon. When the phenoxide behaves like a weak nucleophile and attacks the C4 carbon atom of the BL, the alkyl-oxygen bond scission takes place and respective 3-phenoxybutyrate **1** is formed. As a consequence of this, polymer chains with 3-phenoxybutyrate initial groups and carboxylate growing species are formed. 

The chains with hydroxyl initial groups observed in NMR and ESI-MS spectra are formed when the phenoxide reacts like an alkoxide, i.e., by the addition of a phenoxide anion to the lactone with the ring opening in the acyl-oxygen position, according to the nucleophilic substitution mechanism at the carbonyl carbon (C2) giving unstable *β*-alkoxide ester **2**. After the elimination reaction phenyl crotonate **3** and sodium hydroxide were formed [28,33,41]. Presence of phenyl crotonate in the filtrate after polymer isolation was confirmed by the GC-MS technique (see Figure S1 in Supplementary). Then, NaOH reacts with the next BL molecule. As a result of the hydroxyl anion attack on the C2 acyl carbon, proceeding with the acyl-oxygen bond scission, an unstable intermediate product, *β*-hydroxyester alkoxide **4**, is formed. Next in the acid-base reaction, 3-hydroxybutyric acid salt **5** is formed. Such a salt can partially eliminate water with formation of crotonic acid salt. The obtained sodium salts of 3-hydroxyacid and crotonic acid initiate further polymerization according to the mechanism of polymerization with weak nucleophiles and macromolecules with hydroxyl and crotonate initial groups, and carboxylate propagation centers are present in the reaction mixture. 

Crotonate initial groups may also be formed when the phenoxide behaves as a Brønsted base, i.e., it abstracts the acidic proton of a monomer methylene group with formation of lactone enolate, which undergoes an elimination reaction to form a crotonate anion, initiating further polymerization. The *α*-deprotonation reaction generates a phenol, whose presence was confirmed by GC-MS (see Figure S1 in Supplementary). However, the formation of the crotonate end groups, as a result of the chain transfer to the polymer occurring according to the E1cB mechanism, has to also be taken into account. In BL AROP, crotonate end-groups in polyester are usually present and their quantitative elimination seems to be almost impossible.

The growing species in the investigated processes were carboxylate ones, independent of the phenoxide used. However, the ratio of the respective initial group in the polymer strongly depends on the nucleophilicity and basicity of the initiator.

**Figure 4:**
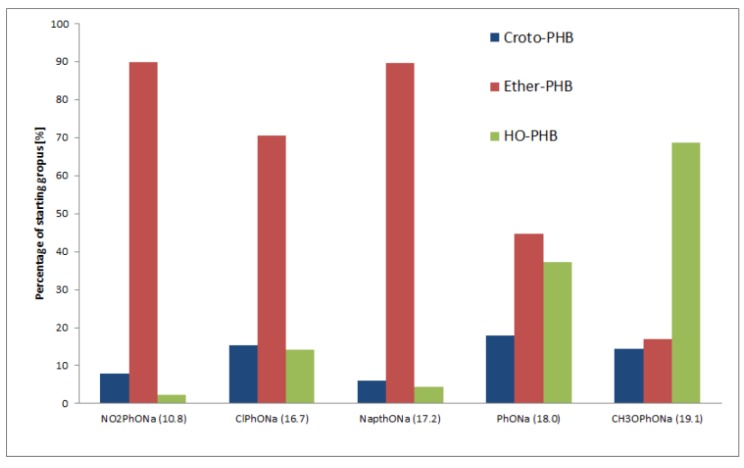
Ratio of crotonate, phenoxy and hydroxy initial groups in the polymer depending on the initiator used (estimated from ^1^H NMR measurements; pKa values presented in parenthesis correspond to phenols used [38]).

The basicity of the initiators varied across a medium range, as all initiators were synthesized from phenol derivatives with a pKa from 10.2 (*p*-nitrophenol) to 19.1 (*p*-methoxyphenol). The second important feature of these phenoxides is their nucleophilicity, which is responsible for the nucleophilic attack at the C2 or C4 carbon of the *β*-butyrolactone ring respectively. Therefore, the order of the basicity/nucleophilicity (*p-NO_2_PhO^−^< p-ClPhO^−^ < NapthO^−^ < PhO^−^ < p-CH_3_OPhO^−^*) of initiators used is important to discuss further results. It was found that the ratio of respective initial groups in obtained polymers strongly depends on the basicity/nucleophilicity of the initiator used (Figure 4). 

The presented results indicate that depending on the sodium phenoxide derivatives used as initiators in BL polymerization, the attack at the C4 carbon of the monomer mainly occurs with formation of polymer chains, with 89.8% of 3-phenoxybutyrate initial groups. Low content of polymer chains with crotonate as the initial group (7.9%) is probably due to the monomer elimination reaction and chain transfer to the polymer, both proceeding according to the E1cB mechanism. Observed traces of hydroxyl groups (2.3%) result from the attack of an initiator at the carbonyl carbon (C2) of BL (see Scheme 1). Results showed that increasing nucleophilicity and basicity of the initiator increased the number of initial hydroxyl groups derived from the initiator attack on C2 carbon at the cost of decreasing the amount of phenoxy groups. In the case of polymerization initiated with *p*-methoxyphenoxide (the most nucleophilic initiator), the resulting polymer possesses 67.2% of chains with 3-hydroxybutyrate initial groups and only 15.9% of phenoxybutyrate ones. Moreover, the increase of initiator basicity affects the *α*-deprotonation reaction and increases the content of polyester crotonate groups up to 17%. A further increase of initiator basicity results in the entire *α*-deprotonation reaction, and the polymer formed possesses only crotonate initial groups [22,26]. 

It is worth noticing that the initiator basicity and nucleophilicity affects only the mechanism and kinetics of the initiation reaction. The kinetics of the chain propagation reaction does not depend on the initiator used, because carboxylate anions are the polymer chain growing species in all described BL anionic polymerizations when the reaction is carried out in the same conditions (solvent, complexing agent, etc.). 

In the BL polymerization initiated with sodium naphthoxide, the results are different from the trend observed for the studied phenoxides, although its basicity is located between *p*-chlorophenoxide and phenoxide. Initial group analysis shows that the naphthoxide anion behaves like a *p*-nitrophenoxide, i.e., the weakest nucleophile and base from the initiators used. It mainly attacks at the C4 carbon of the BL ring. The resulting polymer possesses mainly 3-naphthoxybutyrate initial groups (89.7%) but also a small content of crotonate (6%) and hydroxyl (4,3%) initial groups is observed. Such behavior is probably caused by a steric effect of the naphthyl ring. According to Burgi and Dunitz [42], a nucleophile attacks the carbonyl carbon along a trajectory, where the angle between the nucleophilic attack path and the orientation of the carbonyl group is higher than 90°. The attack of a large naphthoxide anion at carbonyl carbon C2 of BL is very problematic in such a small and strained ring. Therefore, the attack at the alkyl carbon C4 of the ring is preferred, independent of the initiator basicity/nucleophilicity.

## 4. Conclusions

It was found that the mechanism of initiation in anionic polymerization of BL strongly depends on the nucleophilicity and basicity of the initiator used. When strong nucleophiles, e.g., alkali metal methoxide, are applied, the initiation reaction occurs via addition-elimination and the initiator is not incorporated into the polymer chain [32,33,34]. In the case of strong bases, such as superbases or potassium hydride, in the initiation reaction, monomer deprotonation at C2 is definitely the main reaction leading to formation of crotonate acting as the real initiator of the polymerization [22,26]. However, weak nucleophiles and bases such as carboxylate anions initiate the BL polymerization process due to a S_N_2 reaction at the C4 carbon of the monomer. Obtained results of BL polymerization initiated with selected sodium phenoxide derivatives indicate clearly that the structure of the initiator active species does not affect the initiation mechanism. The main factor of the mechanism of *β*-butyrolactone ring opening is definitely the basicity and nucleophilicity of the initiator and phenoxide with the lowest basicity, i.e., the sodium *p*-nitrophenolate reacts mainly like a weak nucleophile. The nucleophile attack mainly occurs at the C4 carbon with formation of 3-*p*-nitrophenoxybutyrate. Analysis of the reaction products also shows generation of small amounts of sodium crotonate and sodium 3-hydroxybutyrate formed in the reaction, where the phenolate behaves as a base (BL α-deprotonation) and strong nucleophile (attack at the carbon C2). Such initiation resulted in formation of macromolecules with phenoxy, hydroxy and crotonate initial groups and carboxylate growing species. In all investigated BL polymerizations initiated with phenoxides, formation of similar polymer end groups was observed, however, the mutual molar ratio of the initial groups significantly depended on phenoxide basicity and nucleophilicity. In the case of the use of sodium *p*-methoxyphenoxide, the most basic, but also the most nucleophilic of the initiators applied in this study, macromolecules possessing hydroxy initial groups predominated. It is important that, depending on the basicity and the nuleophilicity of the initiator used, the initiation reaction in anionic *β*-butyrolactone polymerization proceeds in various ways, but ultimately the centers of chain growth are usually carboxylate ones. Therefore, due to a relatively fast initiation reaction, the growth of the chain determines the rate of polymerization, and under similar conditions, the rate of these polymerizations does not depend on the sodium phenoxide derivative used as the initiator. Naturally, the polymerization rate may be controlled by the counterion diameter and/or polarity of the solvent, which affects the growing species nucleophilicity. Moreover, it is important in BL anionic polymerization to select a proper monomer concentration that is favorable for polymer formation to avoid a base-catalyzed polymer degradation reaction. 

## Figures and Tables

**Table 1:** Results of BL polymerization initiated with selected sodium phenoxides ^a^.

Entry ^b^	Initiator	Mn,_th_^d^ [g·mol^−1^]	Mn,_NMR_^e^ [g·mol^−1^]	Mn,_SEC_ [g·mol^−1^]	Đ
1	sodium *p*-nitrophenoxide	1100	1350	1300	1.19
2	sodium *p*-chlorophenoxide	1100	1150	1100	1.25
3	sodium 1-naphtoxide	1100	800	800	1.22
4	sodium phenoxide	1100	900	800	1.21
5	sodium *p*-methoxyphenoxide	1100	850	950	1.13
6	sodium *p*-nitrophenoxide	10000	3400	3800	1.62
7	sodium *p*-chlorophenoxide	10000	3050	4400	1.62
8	sodium 1-naphtoxide	10000	3000	4100	1.76
9	sodium phenoxide	10000	2400	4600	1.90
10	sodium *p*-methoxyphenoxide	10000	3100	4100	1.78
11 ^c^	sodium phenoxide	10000	7200	10200	1.27
12 ^c^	sodium *p*-nitrophenoxide	10000	7000	9100	1.21

^a^ Polymerizations were carried out in DMSO solvent (BL concentration was 1mol/L) at room temperature up to 100% conversion of monomer; ^b^ Polymerization time: entries 1–5: 2 days, entries 6–10: 14 days and entries 11–12: 5 days; ^c^ Monomer concentration—8 mol/L; ^d^ Calculated form equation: M_n_th = [M]_0_ × 86/[I]_0_ where [M]_0_ and [I]_0_—monomer and initiator concentration, 86—molar mass of BL; ^e^ Estimated form ^1^H NMR from equation: M_n_NMR = (***I***_polymC**H**_ × 86)/(***I***_PhOC**H**-_+ ***I***_HOC**H**_ + ***I***_=C**H**_).

## Supplementary Materials

The following are available online at https://www.mdpi.com/2073-4360/11/7/1221/s1, Figure S1: GC-MS spectrum of filtrate obtained via AROP of BL initiated with sodium phenoxide after polymer precipitation.
