# Supplementary material for: Anionic Polymerization of β-Butyrolactone Initiated with Sodium Phenoxides. The Effect of the Initiator Basicity/Nucleophilicity on the ROP Mechanism
Source: Polymers (Basel). 2019 Jul 22;11(7):1221. doi: 10.3390/polym11071221 (PMC6680796; doi:10.3390/polym11071221)
Supplement: Supplementary file 1 [file polymers-11-01221-s001.pdf]

**Supplementary Materials for**

# Anionic polymerization of β-butyrolactone initiated with sodium phenoxides. The effect of the initiator basicity/nucleophilicity on the ROP mechanism

**Adrian Domiński[1], Tomasz Konieczny[1], Magdalena Zięba[1], Magdalena Klim[1], Piotr Kurcok[1,2]***

[1] *Centre of Polymer and Carbon Materials, Polish Academy of Sciences, 34, M. Curie-Sklodowskiej St., 41-819 Zabrze, Poland*; adrian.dominski@cmpw-pan.edu.pl (A.D.); tomasz.konieczny@cmpw-pan.edu.pl (T.K.); magdalena.zieba@cmpw-pan.edu.pl (M.Z.); klim.magdalena@gmail.com (M.K.); piotr.kurcok@cmpw-pan.edu.pl (P.K.)
[2] *Faculty of Mathematics and Natural Science, Jan Dlugosz University in Czestochowa, 13/15, Armii Krajowej Ave., 42-200 Czestochowa, Poland*
***** Correspondence: *piotr.kurcok@cmpw-pan.edu.pl*

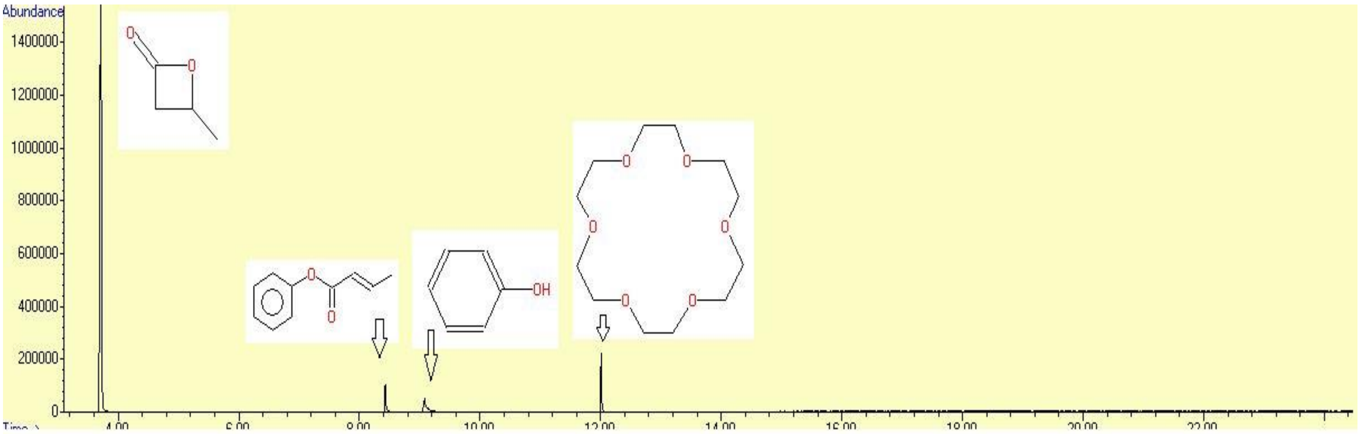


**Figure S1.** GC-MS spectrum of filtrate obtained *via* AROP of BL initiated with sodium phenoxide after polymer precipitation
